# The Therapeutics of Gynæcology and Obstetrics

**Published:** 1880-02

**Authors:** 


THE THERAPEUTICS OF GYNÆCOLOGY AND OBSTETRICS. Comprising the Medical, Dietetic and Hygienic Treatment of Diseases of Women, as set forth by distinguished cotemporary specialists. Edited by Wm. B. Atkinson, A.M., M.D., author of "Hints in the Obstetric Procedure," Lecturer on Diseases of Children at Jefferson Medical College, etc., etc. D. B. BRINTON, Philadelphia, Publisher. 1880.

The "Medical" and the "Surgical Therapeutics" have preceded this, the "Gynæcological Therapeutics," and collectively they embody a series of publications originally projected by Dr. George H. Napheys. The two former works have been sufficiently long before the public to speak their own recommendations. This, the posthumous work of the lamented author, is fully up to the measure of merit that has characterized its predecessors. The therapeusis of diseases of women covers a wide field, and none there are so erudite but that a handy book of reference on this subject will usually be found very convenient. For such purposes is the above work intended, and we feel safe in recommending it to fill the general want.

